# Supplementary material for: Genome Analysis of Thinopyrum intermedium and Its Potential Progenitor Species Using Oligo-FISH
Source: Plants (Basel). 2023 Oct 27;12(21):3705. doi: 10.3390/plants12213705 (PMC10650893; doi:10.3390/plants12213705)
Supplement: Supplementary file 1 [file plants-12-03705-s001.zip › plants-2647541-supplementary.pdf]

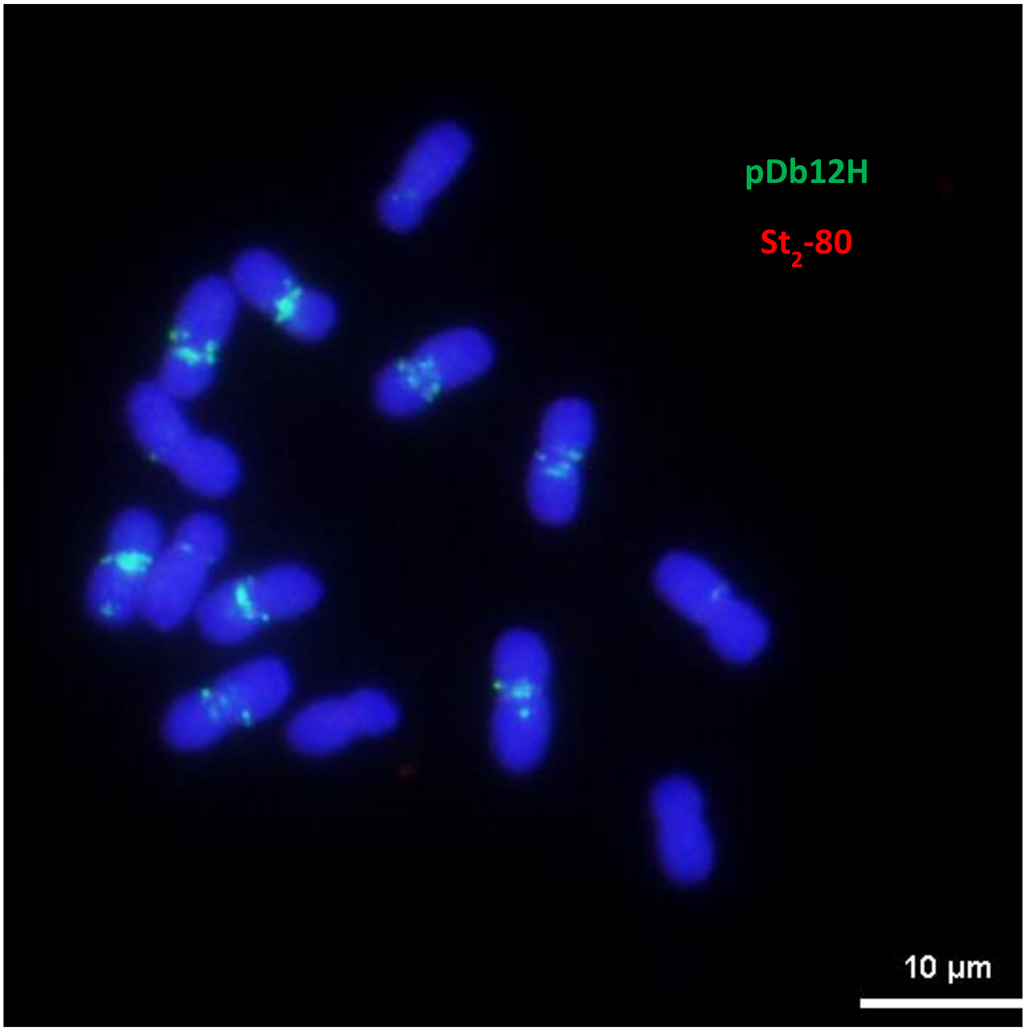

Supplementary Figure S1. Oligonucleotide florescence *in situ* hybridization of chromosomes in *Dasypyrum villosum*. Probes are pDb12H (green) and St_2-80 (red).
